# Bisulfite Sequencing Reveals That *Aspergillus flavus* Holds a Hollow in DNA Methylation

**DOI:** 10.1371/journal.pone.0030349

**Published:** 2012-01-20

**Authors:** Si-Yang Liu, Jian-Qing Lin, Hong-Long Wu, Cheng-Cheng Wang, Shu-Jia Huang, Yan-Feng Luo, Ji-Hua Sun, Jian-Xiang Zhou, Shu-Jing Yan, Jian-Guo He, Jun Wang, Zhu-Mei He

**Affiliations:** 1 MOE Key Laboratory of Aquatic Product Safety, Key Laboratory of Gene Engineering of the Ministry of Education, School of Life Sciences, Sun Yat-sen University, Guangzhou, China; 2 BGI-Shenzhen, Shenzhen, China; UCLA-DOE Institute for Genomics and Proteomics, United States of America

## Abstract

*Aspergillus flavus* first gained scientific attention for its production of aflatoxin. The underlying regulation of aflatoxin biosynthesis has been serving as a theoretical model for biosynthesis of other microbial secondary metabolites. Nevertheless, for several decades, the DNA methylation status, one of the important epigenomic modifications involved in gene regulation, in *A. flavus* remains to be controversial. Here, we applied bisulfite sequencing in conjunction with a biological replicate strategy to investigate the DNA methylation profiling of *A. flavus* genome. Both the bisulfite sequencing data and the methylome comparisons with other fungi confirm that the DNA methylation level of this fungus is negligible. Further investigation into the DNA methyltransferase of *Aspergillus* uncovers its close relationship with RID-like enzymes as well as its divergence with the methyltransferase of species with validated DNA methylation. The lack of repeat contents of the *A. flavus*' genome and the high RIP-index of the small amount of remanent repeat potentially support our speculation that DNA methylation may be absent in *A. flavus* or that it may possess *de novo* DNA methylation which occurs very transiently during the obscure sexual stage of this fungal species. This work contributes to our understanding on the DNA methylation status of *A. flavus*, as well as reinforces our views on the DNA methylation in fungal species. In addition, our strategy of applying bisulfite sequencing to DNA methylation detection in species with low DNA methylation may serve as a reference for later scientific investigations in other hypomethylated species.

## Introduction

*Aspergillus flavus* first came to notoriety for its production of aflatoxin (AF), the most potent naturally occurring toxin and hepatocarcinogenic secondary metabolite [1], [2]. The biosynthesis of AF has been a hot issue among groups of microbiologists, and the underlying regulation pathways have been serving as a theoretical model for biosynthesis of other microbial toxins [3].

A 70 kb gene cluster in chromosome III of *A. flavus* has been discovered to be involved in most of the bioconversion steps in the AF biosynthetic pathway [4]. Some genes located outside the gene cluster have also been found to play a part in AF biosynthesis [5], [6]. Furthermore, some environmental elements, including light, carbon source, nitrogen source, temperature, pH, and antioxidants, are also related to AF biosynthesis [7], [8]. Epigenetic modification, which is considered to be the connection between genotype, phenotype, and environment in most eukaryotes, has been suggested to be an important control mechanism used by fungi to modulate the transcription of genes involved in secondary metabolite production [9]. However, it is still unknown whether the AF biosynthesis process is related to epigenetic variation, especially to DNA methylation.

Although whether DNA methylation exists in *A. flavus* remains controversial, a few pieces of evidence have lent strong positive support. Firstly, when treating *A. flavus* with the DNA methyltransferase inhibitor 5-azacytidine (5-AC), we observed a fluffy phenotype of the colony and a sharp decline in AF production (laboratory work, in submission), suggesting a potential role of DNA methylation in the regulation of AF biosynthesis. Subsequently, we identified a homolog of DNA methyltransferase gene in the *A. flavus* genome and further confirmed its expression throughout the *A. flavus* lifecycle. Furthermore, through high performance liquid chromatography (HPLC) and two-dimensional thin-layer chromatography (2D-TLC), Gowher *et al.* showed that 5-methylcytosine (5 mC) is present in the DNA from *A. flavus* and that the relative amount of 5 mC to total cytosine is approximately 1/400 [10].

On the other hand, a few reports have cast doubt on the existence of DNA methylation in this fungus. First of all, when we applied CpG methylation-sensitive restriction endonucleases (MSREs) *Msp*I/*Hpa*II to *A. flavus*, we could not detect any 5 mC, consistent with previous studies in other *Aspergillus* members [11]. Secondly, 5 mC could not be detected in *Aspergillus* members by 2D-TLC, MSREs digestion, southern hybridization, and immunoblotting [11]–[14]. Finally, no other positive report on 5 mC of *A. flavus* has been released since HPLC and 2D-TLC analysis were first applied to *A. flavus* DNA methylation detection a decade ago [10].

Considering the controversial existence of DNA methylation in *A. flavus*, we chose the bisulfite sequencing (BS-Seq), which is considered as the gold standard for DNA methylation profiling [15], [16], to reveal for the first time a clear and comprehensive DNA methylation profile of *A. flavus*. BS-Seq makes DNA methylation calls in a single-base resolution and in a quantitative way. It not only estimates the global DNA methylation level of *A. flavus*, but also reveals the DNA methylation pattern in different sequence context, thereby providing important insights into the distribution and function of DNA methylation in *A. flavus*. In addition, DNA contamination, if any, can be sensitively detected.

Despite these advantages of BS-Seq, we also noticed the unique disadvantage of this technology—the false positive methylcytosine calls due to the non-conversion of unmethylated cytosines. Therefore, a biological replicate strategy was applied and a false-positive control was set in our experiment.

To further explore the DNA methylome in *A. flavus*, we also compared the BS-Seq data of *A. flavus* and all the other fungi with available data for DNA methylation levels and patterns. Also, we analyzed the divergence of DNA methylatransferases and the genomic features relevant to DNA methylation, such as RIP-index and the repeat components among *A. flavus*, the other *Aspergillus* members, and the species whose methylome has been already investigated. The comparisons, together with the BS-Seq results, reveal that *A. flavus* holds a hollow in DNA methylation. This raises two possible definitions of the DNA methylation status of *Aspergillus* members: they may completely lack DNA methylation; or there may be extremely transient methylated states during the obscure sexual stage of the life cycle, but the process is so evanescent that we can hardly reproducibly determine the DNA methylation status of the cytosines within the genome.

Altogether, this work contributes to our understanding on the DNA methylation status of *A. flavus*, a typical fungal model on secondary metabolisms and reinforces our views on the DNA methylation in fungal species. Furthermore, our strategy of applying BS-Seq to DNA methylation detection in species with low DNA methylation may serve as a reference for future scientific investigations in other hypomethylated species.

## Results

### Bisulfite Sequencing Reveals a Lack of DNA Methylation in *A. flavus*

BS-Seq of two biological replicates of *A. flavus* NRRL 3357 was carried out. In total, each biological replicate yielded 1.2 Gbp raw data. For each of them, approximately 90% of the total reads were unambiguously mapped to the genome, covering 91% of the 19.3 million cytosines within the *A. flavus* genome with at least 1 read. The effective average sequencing depth per strand reached 14× for both replicates. Both libraries showed nearly complete bisulfite conversion (>99.5%). In addition, we observed a very high concordance of the sequencing depth across cytosines between the two biological replicates (Pearson Correlation Efficient = 0.98), indicating the unbiased construction of both libraries and the practicability of our overlapping methods for methylcytosine determination (see below). Processing of the data and quality control procedures were described in the Materials and Methods section and data production is summarized in Table S1 and Figure S1.

The global DNA methylation level of *A. flavus* NRRL 3357 was estimated by the amount of virtually methylated cytosines as well as the non-converted unmethylated cytosines divided by the total amount of cytosines detected in each sample. The global level was low in both biological replicates of this fungal species, ranging from 0.44% (replicate 1) to 0.47% (replicate 2), which is comparable to the DNA methylation level of the unmethylated lambda DNA in the corresponding base context (Table 1).

**Table 1:** Global DNA methylation level of different types of cytosine.

Types of cytosines	C	CG	CA	CT	CC
*A. flavus* Replicate 1	0.00465	0.00430	0.00664	0.00378	0.00364
*A. flavus* Replicate 2	0.00465	0.00430	0.00663	0.00378	0.00366
Control Replicate 1	0.00443	0.00431	0.00623	0.00341	0.00334
Control Replicate 2	0.00448	0.00437	0.00605	0.00350	0.00361

However, global DNA methylation levels are only an indirect indicator of the methylcytosines in a genome. For example, the silkworm possess approximately10,000 highly conserved methylcytosines, but its global DNA methylation level merely ranges from 0.2% to 0.7% [17].This seemingly contradictory observation is due to the clustering of highly methylated methylcytosines within a very limited number of regions. Taken this into consideration, we launched an investigation on the DNA methylation status of each cytosine in *A. flavus* genome.

**Figure 1:**
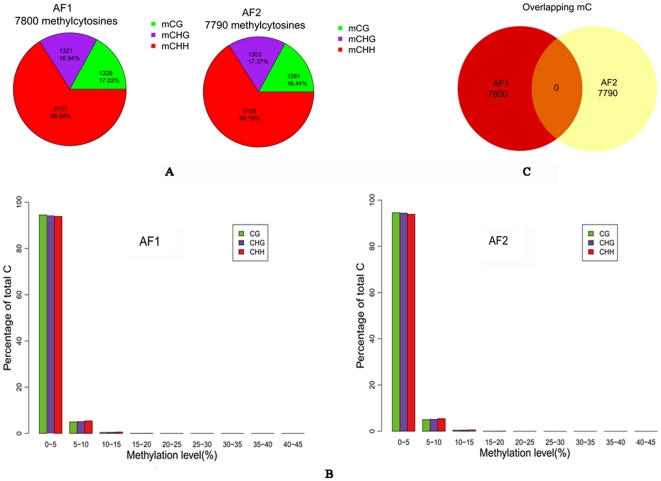
Bisulfite Sequencing Reveals a Lack of DNA Methylation in *A. flavus.* **A.** Number of methylcytosines passed the binomial correction algorithm in different contexts for each of both *A. flavus* biological replicates AF1 and AF2. **B.** DNA methylation levels of the methylcytosines in each biological replicate. **C.** Venn figure showing no methylcytosine is detected in both *A. flavus* biological replicates.

We first applied a correction algorithm based on the binomial model for 5 mC detection [18] (see Materials and Methods section for details). At first, 7,800 5 mC (replicate 1) and 7,790 5 mC (replicate 2) were detected, taking up nearly 0.04% of the total genomic cytosines and most of detected methylcytosines were located in the CHH or CHG context (Figure 1A). Nevertheless, DNA methylation levels of the detected 5 mC were low (Figure 1B) — only a very small fraction of them were above 5% and few of them were higher than 15%. We suspected that these methylcytosines are non-converted unmethylated cytosines rather than true methylcytosines. Therefore, in order to persuasively determine the true methylation state of these cytosines and to increase the reproducibility of our conclusions, we applied an overlapping strategy and required the true 5 mC to be the 5 mC detected in both biological replicates. This time, no 5 mC was detected (Figure 1C).

Further validation applying traditional bisulfite-PCR and sequencing (BS-PCR) for 3 selected batches of sequences, including 24 methylcytosines detected by any one of the two replicates, confirmed that these 5 mCs were indeed unmethylated. All the primers for BS-PCR validation were detailed in Table S2.

Although *A. flavus* possesses a DNA methyltransferase gene homolog actively expressed throughout the life cycle (data not shown) and it has been reported to possess 1 methylcytosine out of 400 cytosines within the genome [10], our BS-Seq data clearly demonstrate the negligible level of methylation in this fungal species (further discussed in the Discussion section). Furthermore, through comparing the DNA methylation profiling of *A. flavus* with that of other 5 fungi, including *Phycomyces blakesleeanus*, *Coprinopsis cinerea*, *Laccaria bicolor*, *Postia placenta*, and *Uncinocarpus reesii*, revealed by Zemach and Ziberman with the same technology, which shows low but sufficiently significant DNA methylation (2%–6%, mC/total C) [19], we found that *A. flavus*, like the *Saccharomyces* members, locates in the breakpoint during the evolutionary course of DNA methylation in fungal species.

### DNA Methyltransferases of *Aspergillus* Members Show Significant Divergence Compared with Methylated Species

**Figure 2:**
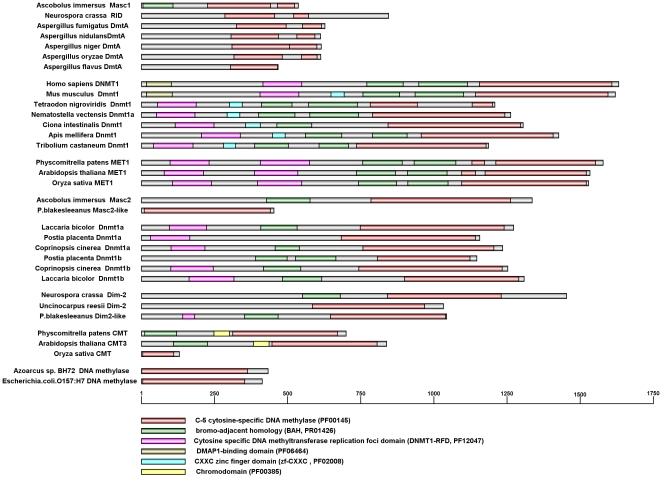
Structure of DNA methyltransferase proteins. The conserved domains of DNA methyltransferases from 12 fungi, 2 mammals, 5 invertebrates, and 3 plants. The DNA methylase domains locate on the N-terminal regions of each protein. The protein domain architectures were generated using the protein domain visualization software DOG 2.0 [48] based on the domain limits Pfam [47] with help of InterProScan [46].

Since the occurrence of DNA methylation relies on DNA methyltransferase, the enzyme family that catalyzes the transfer of the methyl group to DNA, we wonder whether there is some special characteristics of the DNA methyltransferase homolog within the *A. flavus* genome, which exhibits negligible DNA methylation levels, compared with the DNA methyltransferases of the species with hypermethylated genomes. Therefore, we analyzed the conserved DNA methylase domain of the 6 fungi as well as several other DNA methyltransferases from the selected 7 fungi, 2 mammals, 5 invertebrates, and 3 plants (Figure 2) and depicted a phylogenetic tree based on the conserved domain of the DNA methyltransferases.

**Figure 3:**
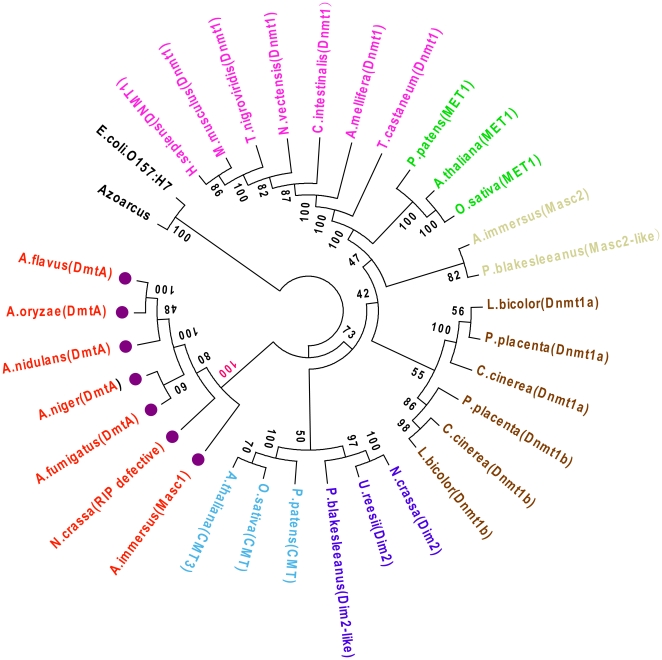
Phylogenetic tree of DNA methyltransferase. This tree intends to represent the divergence between DNA methyltransferases of *Aspergillus* members and those displaying DNA methyltransferase capabilities. It was constructed using Neighbor-joining statistical methods based on the Poisson model. The C-5 cytosine-specific DNA methylase conservative domains were identified based on the domain limits Pfam [47] with help of InterProScan [46]. The branches with bootstrap value <80 should be controversial due to lack of statistically robustness and we don't discuss these branches in the main text.

It seems to us the character of the DNA methyltransferase possessed by one species turns out to be a very effective predictor of its DNA methylation status. Of our interest, we find that the DNA methyltransferases possessed by the *Aspergillus* members are closely related to the repeat induced point-mutation defective (RID) of *Neurospora* and the Masc1 of *Ascobolus immerses*, the two DNA methyltransferase homologs that have not been proven to have DNA methylase catalysis capability [20] (Figure 3 highlighted in red). These groups of DNA methyltransferase are highly divergent from the DNA methyltransferases that occur in the species with validated DNA methylation (bootstrap value: 100). So far, it seems logical to infer that the DmtA occupied by the *Aspergillus* member*s* might not be a true DNA methyltransferase, but may possibly be an enzyme responsible for repeat induced point-mutation (RIP).

### Lack of Repeat Content in *Aspergillus* and the Repeats Display Significantly Higher RIP-index

**Figure 4:**
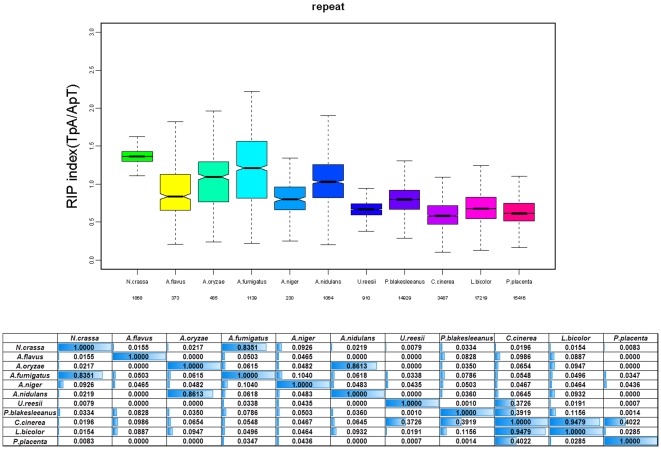
RIP index of the fungal repeats. The upper boxplot demonstrates the RIP index of *N. crassa*, 5 *Aspergillus* members and the other 5 fungi with available BS-Seq data. *Aspergillu*s members consistently display lower RIP-index than *N. crassa* but higher RIP-index than the other 5 fungi without RID-like homologs. The lower table denotes the p-value of two-tailed t-test between two fungi.

Despite the fact that DNA methylation has only been observed in the gene body of the active genes of the *U. reesii* [19], DNA methylation has long been considered as an important genome defense strategy for fungi. Consistent with this fact, all the other 5 fungi investigated by BS-Seq showed heavy DNA methylation in their repeat regions [19]. However, our study showed that *A. flavus* lacked DNA methylation. We annotated a whole set of repeats within the genome of *A. flavus* and expectedly, *A. flavus* exhibited a statistically significant lack of repeat components compared with the other fungi (chi-square test, p-value<1.85e-86). In order to provide insights into the potential function of the RID-like enzyme in *Aspergillus* members, we applied the formula described in [21] and compared the RIP-index of the repeat regions among *N. crassa*, 5 *Aspergillus* members and the other 5 fungi mentioned above (Figure 4). Although the RIP-index distribution of *N. crassa* is globally higher than all of the rest fungi, we found that the RIP-index of the repeat regions of *A. flavus* and other *Aspergillus* members was significantly higher than that of the other 5 fungi with no RID-like homologs in their genome (t-test, p-value<0.1). Our results indicated that RIP, which was first discovered in *Neurospora* [21], [22] and later observed in *A. niger* [23], might potentially work in some of the *Aspergillus* members, including *A. flavus*, whose sexual state has been discovered [24] (further discussed in the Discussion section).

## Discussion

Epigenetic markers serve as a bridge between genetic components and the environment. DNA methylation is one of these markers and plays a vital role in the regulation of gene expression in eukaryotes. Several physiological processes, such as embryogenesis, genomic imprinting, X-inactivation, tumorigenesis in mammals [25], [26], transposon silencing in plants [27], and genome defense in fungi [28], have been linked to the modification of a methyl-group into the cytosine of DNA. However, this genomic modification is not present in all eukaryotes. While the DNA methylation level is high in mammals and plants, this level is very scarce among insects [17], [29]–[31] and varies significantly in fungal species [11]. Most fungi have varying low levels of DNA methylation, ranging from imperceptible to just barely detectable [11], [32].

Whether DNA methylation exists in *Aspergillus* remains controversial. The *Aspergillus* member *A. nidulans* does not show any observable difference between *Msp*I and *Hpa*II restriction patterns. However, high-frequency conversion to a “fluffy” developmental phenotype could be observed when this fungus was treated by DNA methyltransferase inhibitor 5-AC [12]. Our manipulations confirm the above phenomena in *A. flavus* and our laboratory also discovered that the production of AF declines when treating *A. flavus* with 5-AC. Additionally, although *A. flavus* has been reported to contain 0.25% methylcytosines within its genome [10], there are no other positive reports on the DNA methylation of this fungus and no specific methylated gene in *A. flavus* has been identified since then.

Indeed, sensitive and accurate detection of 5 mC relies largely on the technology and the experimental strategy for DNA methylation analysis. Methods based on the use of MSREs lack resolution and they can only detect 5 mC in the CG context. Thus, they are not suitable methods for the DNA methylation profiling of species with low levels of DNA methylation or with DNA methylation in cytosines in the non-CG context [33]. HPLC is relatively sensitive in the detection of global levels of DNA methylation but it tells nothing about the sequence context of the 5 mC and the distribution of the 5 mC [33]. It is also hard for a single HPLC experiment to determine whether the DNA sample has been contaminated by heterogeneous DNA fragments such as bacteria DNA. This can severely confuse the DNA methylation detection of species with low DNA methylation levels like *A. flavus*.

Curious to see if DNA methylation is acting to regulate the production of AF in this fungal species, we applied BS-Seq for the detection of DNA methylation in *A. flavus*. Also, we set two biological replicates for BS-Seq to guarantee a robust conclusion and applied BS-PCR to validate our BS-Seq results. Unexpectedly, our results reveal the DNA methylation level of *A. flavus* to be negligible. The zero methylcytosines that can be detected by two biological replicates and the failure of BS-PCR to reproduce the methylcytosines that were detected by only one replicate suggests two possibilities: First, DNA methylation pathway lost early in *A. flavus*. This can be supported by the lack of repeat content in *A. flavus* genome and if this fungus is asexual throughout the duration of its life, which is an important source for repeat amplification [34], it is understandable that loss of DNA methylation did not act up to negative selection. The second possibility is that *de novo* DNA methylation might occur transiently during a life phase and it is hard for us to detect any methylcytosines from the fungi reproducibly. If this turns out to be the case, we suspect the life phase when DNA methylation occurs may most likely be the obscure sexual stage of this fungus [24].

Comparison of the DNA methyltransferases between *A. flavus* with the other selected species that are hypermethylated in their genome reveals that the DNA methyltransferase of *A. flavus* as well as the other *Aspergillus* members is closely related to the Masc1 in *A. immersus* and the RIP defective in *N. crassa* [20], both of which were defective in transferring methyl-groups into the cytosines of DNA. So far, we are not sure whether this RID-homolog, which is actively expressed throughout the life cycle of *A. flavus* but is not an effective DNA methyltransferase, is engaged in the RIP process of the asexual fungus *Aspergillus*. However, repeat sequences of *Aspergillus* show higher RIP-index compared with those that do not possess RIP genome defense system. Since RIP has been reported to occur in two asexual fungi [35], it is logical to predict that in *A. flavus*, RIP also exists and may even be accompanied by DNA methylation during the very transient and obscure sexual stages [20].

Whatever the true status of the DNA methylome of *A. flavus* may be, DNA methylation in *A. flavus* is at such a low level that it can hardly be the target of the DNA methyltransferase inhibitor 5-AC. Furthermore, there is little possibility that DNA methylation acts to regulate the expressions of the genes involved in AF pathways. Therefore, we propose that there may be other mechanisms other than DNA methylation leading to the phenotype changes and AF reduction when *A. flavus* is treated with 5-AC. Mechanisms concerning genomic histone modification [36], [37] or the DNA mutagen effect of 5-AC [38] are possibilities.

Furthermore, the RIP-index of the repeat of *A. flavus* turns out to be higher than the fungi without RID-like enzyme, suggesting this fungus may possibly possess RIP process during the obscure sexual-stage which is very evanescent and may be potentially related to DNA methylation.

## Materials and Methods

### Data Availability

The raw Illumina sequencing data are deposited in GEO (http://www.ncbi.nlm.nih.gov/geo/) at NCBI with the accession number GSE32177.

### Fungal Strain and Culture Conditions

*A. flavus* NRRL 3357, a producer of aflatoxins B1, B2, G1, and G2, was cultured in liquid medium of 2.4% (w/v) potato dextrose broth (PDB, purchased from Difco) at 200 rpm. All cultures were kept at 30°C and kept away from light to avoid its effect on AF biosynthesis.

### DNA Extraction

Genomic DNA was extracted from *A. flavus* mycelia using a modified method as described on the web site of Integrated Fungal Research Center (http://www.aspergillusflavus.org/protocols/). A total of 2 g of mycelia, obtained through filtered culture, was grinded to a fine powder in liquid nitrogen. The powder was promptly scooped into a 10 ml tube containing 2 ml phenol and 4 ml H buffer (10 mM Tris-HCl, pH 7.5; 100 mM LiCl; 10 mM EDTA; 0.5% SDS), and vortexed thoroughly. The suspension was incubated at 60°C for 5 min and cooled on ice before 2 ml chloroform: isoamyl alcohol (24∶1, v/v) was added. After centrifugation at 11,000 rpm in 4°C for 10 min, the aqueous layer was transferred into a fresh tube. A total volume of 4 ml phenol and 4 ml chloroform: isoamyl alcohol (24∶1, v/v) were used successively to purify DNA. The aqueous layer was transferred into a fresh tube. NaAc and isopropanol were added to a final concentration of 0.3 M and 50% (v/v), respectively, mixed gently, and placed in a −20°C freezer for at least 45 min for precipitation. After centrifugation at 11,000 rpm in 4°C for 10 min, the precipitate was resuspended in 2 ml TE buffer containing 10 µg/ml protease K and incubated in 37°C for over 60 min. Another 2 ml TE was added before 4 ml phenol and 4 ml chloroform: isoamyl alcohol (24∶1, v/v) were used successively to purify DNA. RNase I was added into the aqueous layer collection with a final concentration of 10 µg/ml and then incubated at 37°C for at least 60 min. A volume of 4 ml chloroform: isoamyl alcohol (24∶1, v/v) were used several times to remove the impurities completely. NaAc and isopropanol were added into the aqueous layer collection to a final concentration of 0.3 M and 50% (v/v) respectively, mixed gently, and placed in −20°C freezer for at least 1 h for precipitation. The mixture was then centrifuged for 10 min at 11,000 rpm in 4°C. After washing with 70% ethanol twice, the precipitated DNA was resuspended in 100 µl TE buffer. The concentration and purity of DNA was determined by the measurement of A_260_ and A_280_ using a NanoVue spectrophotometer (GE Healthcare).

### BS Library Construction and Bisulfite Sequencing of *A. flavus*

The overall workflow was summarized in Figure S2.

An unmethylated DNA fragment (48,502 bp) isolated from infected *E. coli*, was used to determine the bisulfite conversion efficiency and as a quality control for bisulfite treatment.

Before bisulfite treatment, 25 ng λ-DNA were added to the 5 µg genomic DNA of *A. flavus*. Then the mixed DNA was fragmented with a Sonicator (Sonics &Materials) to a mean size of approximately 200 bp for biological replicate 1 and around 250 bp for biological replicate 2. After blunt ending, 3′-end addition of dA, Illumina methylated adapters were added according to the Illumina manufacturer's instructions. The bisulfite conversion of *A. flavus* DNA was carried out using a modified NH_4_SO_4_-based protocol [39] and amplified by 12 cycles of PCR. Ultra-high-throughput pair-end sequencing was carried out using the Illumina Genetic Analyzer (GA2) according to the manufacturer instructions. Raw GA sequencing data was processed by Illumina base-calling pipeline (SolexaPipeline-1.0).

### Mapping and Initial Processing of the Data

After removal of the adaptor sequences, the 49 bp reads from each of the biological replicates were aligned to the *A. flavus* genome. Because of the strand specificity of DNA methylation, two rounds of alignments were carried out. All cytosines of the bisulfite-converted reads were transformed to thymine and aligned to the genome sequences termed the “T genome”, whose cytosines had been converted to thymine. Also, all guanine of the bisulfite converted reads were transformed to adenosine and aligned to the genome sequences termed the “A genome” whose guanines had been converted to adenosine. The alignments were carried out with BGI SOAP aligner version 2.01 [40], allowing up to two mismatches for successful mapping. And then, we transformed each aligned read and the two strands of the *A. flavus* genome back to their original forms to build an alignment between the original forms. Cytosines in the BS-Seq reads that are also matched to the corresponding cytosines in the plus (Watson) strand, or otherwise guanines in the Methyl C-seq reads, that are also matched to the corresponding guanines in the minus (Crick) strand will be regarded as potential 5 mCs. Note that for a particular cytosine, it could be in the methylated state (mC) and it could also be in the unmethylated state due to the heterogeneity of the cell populations. We emphasized this here for easier comprehension of the further analysis.

We carried out several filtrations of the data to ensure the accuracy for methylation calls. First, we filtered out all cytosines with an Illumina Q score smaller than 20, which guaranteed that a base was correctly called at more than 99% probability. Second, multiple reads mapped to the same start positions as well as the same end positions were defined as clonal duplicates because these events seldom occur with the sonicated pair-end reads. We filtered these reads to avoid inaccuracy that might be caused by the bias during the PCR amplification. Third, we filtered out the reads that were mapped to multiple sites within the genome. Finally, all the non-clonal reads with sufficient Q scores and that were unambiguously mapped to the genome were applied for further analysis.

Summary of the data quantity after each step of filtration is shown in Table S1 for *A. flavus*. Conversion rate was estimated by (1-the number of methylated cytosines divided by the number of total cytosines of the unmethylated lambda DNA sequences) ×100%.

### Determination of DNA Methylation Level and Identification of 5 mC

DNA methylation level of the genome was calculated as the number of potential 5 mC detected in the preceding section, divided by the total number of identified cytosines within the whole set of reads.

To identify the methylcytosines from the background noise due to non-conversion of the unmethylated cytosines, we first used the methylation level of the unmethylated lambda DNA as the noise control, which provides a measure for the false-positive rate (sum of the nonconversion rate and thymidine-to-cytosine sequencing errors). Then we applied the correction algorithm of [18] and set the significance threshold to be 0.01. The interrogated cytosines were required to be sequenced for at least 10 times to improve the power of this algorithm.
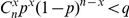
The *p* in the formula is the false-positive rate and the *q* is the significance threshold.

However, since the DNA methylation level of the resulting methylcytosines turned out to be very low and the BS-Seq project of silkworm [17] discovered that even cytosines with high methylation level could still be false positive. Therefore, we applied the same biological replicates overlapping strategy as [17] and searched for the overlapping methylcytosines independently identified in both replicates.

### BS-PCR Validation

We selected three target regions containing 24 methylcytosines identified in only one of the two biological replicates for bisulfite PCR using Sanger sequencing to validate the methylation status. Primers were designed by MethPrimer and listed in Table S2.

One microgram of genomic DNA from *A. flavus* biological replicate 1 was bisulfite-converted following the same protocol as above. We set the target sequence as the subject and the raw reads as the query and applied blastn to align the reads to the target sequences (e-value<1e-3, matched sequences > = 30). 100% of the reads were successfully mapped to the target regions.

### Public Data Used in the Bioinformatic Analysis, Repeat Annotation, and RIP-index Calculation

The databases and the URLs of the public data used in our study were summarized in Table S3.

Repetitive sequences of *A. flavus* as well as the other fungi were annotated by an in-house pipeline of BGI which combines analysis against the Repbase library and *de novo* repeat identification. In brief, we identified known transposable elements using RepeatMasker against the Repbase library (Smit AFA, Hubley R, Green P. RepeatMasker Open-3.0. 1996–2010 <http://www.repeatmasker.org>). Also, we aligned the genome sequence to the curated transposable element-related proteins using to identify highly diverged transposable elements.

Furthermore, we use RepeatScout [41], PilerFinder [42] and TLR Finder [43] to construct a *de novo* repeat library.

RIP index is calculated as the ratio of “TpA/ApT”. Since one premium of RIP is that the homolog section reaches 400 bp [21], [44], [45], we only counted the repeats with length > = 400 bp here.

### Blastp for DNA Methyltransferase Homolog of *A. flavus* Against the DNA Methyltransferases of Other Species

We set the whole set of protein sequences of the *A. flavus* as the query and the protein sequence of the DNA methyltransferase groups of 10 fungi, 3 plants, 6 invertebrates and 2 mammals (summarized in Table S3) as the subject and carried out blastp to search for any homologous sequences (E-value< = 1e-10) of DNA methyltransferase within the *A. flavus* genome.

### Identification of the C-5 Cytosine-specific DNA Methylase Conservative Domain and Construction of Phylogenetic Tree

We use InterProScan [46] to identify the C-5 cytosine-specific DNA methylase conservative domain of all the available DNA methyltransferase protein sequence based on the domain limits Pfam [47]. The protein domain architectures were generated using the protein domain visualization software DOG 2.0 [48]. Since we observed that the fungal DNA methyltransferases have a more similar sequence with DNMT1 than DNMT3, we therefore intended the DNMT1-like DNA methyltransferases families from the other selected species as the investigated targets.

The phylogenetic tree was constructed using MEGA software [49] based on the protein sequences of the conserved C-5 cytosine-specific DNA methylase domain. Both maximum likelihood statistical methods based on the JTT model and Neighbor-joining statistical methods based on the Poisson model were used to analyze the tree and 100 bootstrap replications were used to test the tree. Only branches with a bootstrap value higher than 80 are further discussed in the main texts. Bacterial *E.coli* methyltransferases were used as an out-group. The two statistical methods produced the same topology results. Finally the tree based on Neighbor-joining method was subjected for further embellishment. We also carried out the analysis and applied the whole protein sequences of DNA methyltransferases, which also produced the same topological results.

## Supporting Information

Figure S1 **Accumulating sequencing depth for each biological replicate in different context.** The Y axis denotes the percent of the *A. flavus* genomes that is covered by differing minimum number of reads (Sequencing depth, X axis) in biological replicate 1 (A) and biological replicate 2 (B).
(TIF)

Figure S2 **Overall workflow of BS library construction and bisulfite sequencing.**
(TIF)

Table S1 **Summary of data production.**
(XLSX)

Table S2 **Primers used for BS-PCR.**
(XLSX)

Table S3 **Summary of the public data used in the bioinformatic analysis.**
(XLSX)
