# Supplementary figures and images for: Bisulfite Sequencing Reveals That Aspergillus flavus Holds a Hollow in DNA Methylation
Source: PLoS One. 2012 Jan 20;7(1):e30349. doi: 10.1371/journal.pone.0030349 (PMC3262820; doi:10.1371/journal.pone.0030349)

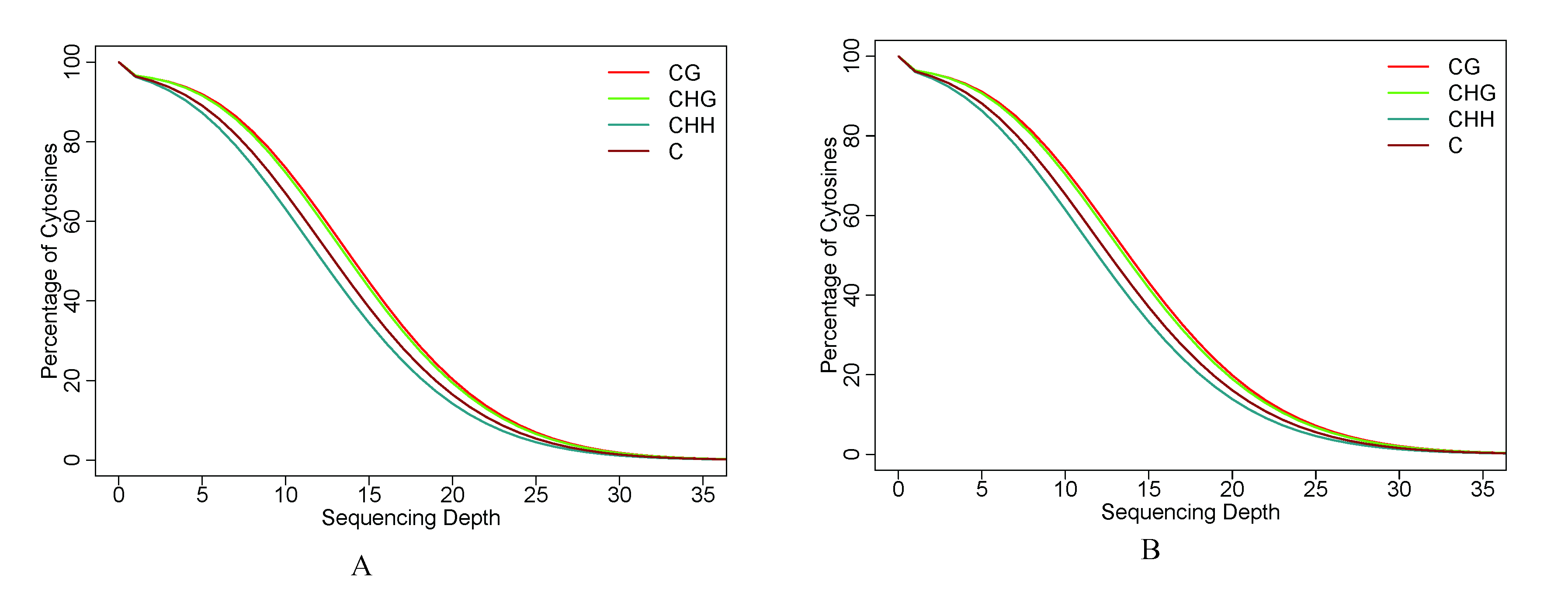

Supplement: Figure S1 — Accumulating sequencing depth for each biological replicate in different context. The Y axis denotes the percent of the A. flavus genomes that is covered by differing minimum number of reads (Sequencing depth, X axis) in biological replicate 1 (A) and biological replicate 2 (B). (TIF) [file pone.0030349.s001.tif]
